# Does Awareness of Aging Matter? The Moderating Function of Awareness of Age-Related Change on the Relationships Between COVID-19 Disruption, Perceived Stress, and Affect

**DOI:** 10.1093/geronb/gbad093

**Published:** 2023-06-20

**Authors:** Elli Kolovos, Tim D Windsor

**Affiliations:** College of Education, Psychology and Social Work, Flinders University, Adelaide, South Australia, Australia; College of Education, Psychology and Social Work, Flinders University, Adelaide, South Australia, Australia

**Keywords:** Emotional well-being, Pandemic, Subjective aging

## Abstract

**Objectives:**

How people reflect on their own age may influence their well-being in the face of disruptions associated with the coronavirus (COVID-19). Subjective aging was operationalized in terms of one’s awareness of age-related change (AARC), specifically, the gains and losses associated with aging. We developed a measure assessing disruptions to daily life associated with the COVID-19 pandemic across 3 dimensions (i.e., Social and Lifestyle Disruption, Work and Health Disruption, and Others Contracting COVID-19). We hypothesized that COVID-19 disruption would be positively associated with both AARC-losses and AARC-gains. Greater COVID-19 disruption would also be associated with poorer psychosocial outcomes (higher perceived stress and negative affect [NA] and lower positive affect [PA]) and these associations would be stronger for those reporting greater AARC-losses and weaker for those reporting greater AARC-gains.

**Methods:**

Cross-sectional questionnaire data were collected from 263 participants from the United States (aged 40–83; mean age: 62.88 years, standard deviation = 9.00; 56.3% females).

**Results:**

After controlling for age, gender, education, employment, socioeconomic status, and physical functioning, greater Work and Health Disruption was associated with greater AARC-losses. Greater Social and Lifestyle Disruption was associated with both greater AARC-gains and AARC-losses. Moderation effects showed an exacerbating effect of AARC-losses on NA in the face of Work and Health Disruption and a protective effect of AARC-gains on PA in the context of Social and Lifestyle Disruption.

**Discussion:**

We extend research detailing antecedents of AARC and highlight the need for longitudinal research that considers the ever-changing nature of the pandemic.

Disruptions associated with the coronavirus (COVID-19) have become significant stressors in many people’s lives and are predicted to have long-lasting impacts on mental health and aging over the next decade (Carpenter et al., 2022). Since the beginning of the pandemic, older adults have frequently been perceived as a homogenous risk group, advised to strictly follow protective measures to prevent the potential for serious illness or death from the virus (Ayalon et al., 2020). Focusing on chronological age as a determinant of COVID-19 risk addresses important public health concerns but fails to recognize the heterogeneity among older adults (Seifert, 2021) potentially resulting in them perceiving themselves as in need, fragile or old (see Stereotype Embodiment Theory; Levy, 2009). We examined older adults’ subjective aging and its associations with psychosocial outcomes experienced in the face of disruptions associated with the COVID-19 pandemic. Specifically, we focused on (a) associations of COVID-19 disruption with awareness of aging, and (b) whether COVID-19 disruption was more strongly associated with psychosocial outcomes (i.e., greater perceived stress and negative affect [NA], and lower positive affect [PA]) among those who reported relatively greater awareness of aging-related losses, and relatively lower awareness of aging-related gains.

## Views on Aging, Subjective Aging, and COVID-19

Views on aging refer to conceptions of older people, old age, and aging in general (Kornadt et al., 2020). Subjective aging is one such conception that represents how one perceives their own age and the aging process (Diehl et al., 2014). Emerging research suggests that subjective aging may influence psychosocial functioning in the context of COVID-19. During the initial stages of the pandemic, an older “felt age” relative to chronological age was associated with greater loneliness (Vitman Schorr et al., 2021), depression, and anxiety (Avidor et al., 2022). Losada-Baltar et al. (2020) found negative self-perceptions of aging to be associated with greater loneliness, psychological distress, and greater reactivity to COVID-19-related stressors. A follow-up study (Losada-Baltar et al., 2021) found support for these associations across four separate time points between March and May 2020. In addition, Yeung et al. (2021) found that positive or “successful” self-perceptions of aging were associated with better affect and methods of coping in the context of COVID-19.

## Subjective Aging as Awareness of Age-Related Change

Diehl and Wahl (2010) presented a multidimensional framework for operationalizing subjective aging in terms of awareness of age-related losses (AARC-losses), which capture perceptions of the undesirable consequences of aging (e.g., functional decline) and awareness of age-related gains (AARC-gains) which reflect perceptions of the positive aspects of growth (e.g., better relationships) associated with aging. Studies have reported associations between AARC-losses and poor psychosocial outcomes such as increased NA (Neupert & Bellingtier, 2017) and a recent meta-analysis (Sabatini et al., 2020) found that while AARC-losses was associated with poorer subjective well-being, AARC-gains was related to better well-being.

## Associations of COVID-19 Disruption and AARC-Gains and AARC-Losses

Diehl and Wahl (2010) proposed a heuristic framework that identified theoretical antecedents of AARC, with current life events specified as one factor that may influence AARC-gains and AARC-losses (see also Rupprecht et al., 2022). More recently, Wahl et al. (2022) proposed that the pandemic may act as a history-graded event influencing AARC over and above normative age-graded changes such as declining physical functioning (Sabatini et al., 2022). Wahl et al. (2022) modeled normative age-graded changes and pandemic-specific changes in AARC-gains and AARC-losses from 2012 to 2020 and reported steeper increases in AARC-gains and AARC-losses after the onset of the pandemic. The authors attributed increases in AARC to the stereotyping of older adults as a homogenous risk group, advised to strictly follow COVID-19 protective measures, further suggesting that for some individuals, negative age stereotypes may trigger a greater focus on their own age-related vulnerabilities, whereas others may be more motivated to dissociate their self-image from old age. Terracciano et al. (2021) found that individuals felt younger during the emergence of the pandemic and that this was particularly true for those who endorsed the belief that “coronavirus is only a threat to older people.”

Few studies have directly measured COVID-19 disruption as a predictor of increases in both AARC-gains and AARC-losses, but rather have inferred these links by measuring subjective aging during the initial phases of the pandemic. Less nuanced unidimensional measures of subjective aging (i.e., “how old do you feel?”) have been used, despite support for a multidimensional conceptualization of both age-related gains and losses (Diehl & Wahl, 2010; Kornadt et al., 2020). The present study will extend existing literature by assessing whether greater COVID-19 disruption is associated with greater AARC-losses and AARC-gains.

## The Moderating Function of AARC

Emerging studies also highlight the possible role of subjective aging as a moderator of psychological distress in the context of COVID-19. For example, during the initial stages of the pandemic, an older subjective age strengthened positive relationships between loneliness and anxiety, depression and peritraumatic distress (Shrira et al., 2020), and COVID-19 health worries and peritraumatic distress (Greenblatt-Kimron et al., 2021).

Yeung et al.’s (2021) finding that perceptions of successful aging (akin to AARC-gains) were associated with adaptive coping strategies in the context of COVID-19 aligns with the theoretical underpinnings of AARC which postulates that AARC-gains and AARC-losses influence psychosocial functioning through the operation of self-regulatory processes (Diehl & Wahl, 2010; Wilton-Harding & Windsor, 2021). Research shows that those experiencing greater AARC-gains (and to a lesser extent lower AARC-losses) may be more flexible in managing goals (Dutt et al., 2018). These individuals may better adapt to disruption via both increased effort and perseverance in pursuing goals (i.e., assimilative coping) or by judiciously adjusting their goals in the face of disruption (i.e., accommodative coping; Brandtstädter & Rothermund, 2002), promoting adaptation and maintenance of psychosocial functioning. Ultimately, the AARC conceptualization of subjective aging not only supports a rationale for moderation but also identifies AARC-losses and AARC-gains as separate dimensions that could affect associations between COVID-related disruption and psychosocial outcomes in different ways.

## The Present Study

We examined (a) associations of COVID-19 disruption with AARC (gains and losses), and (b) whether AARC (gains and losses) moderated associations of COVID-19 disruption with psychosocial outcomes (perceived stress, NA, and PA). COVID-19 disruption represented the extent to which the pandemic has disrupted an individual’s life from March 2020 to when data were collected in July 2021. It was expected that individuals reporting greater COVID-19 disruption would also report greater AARC-gains and AARC-losses. We also expected that associations of COVID-19 disruption with worse psychosocial outcomes (i.e., greater perceived stress and NA, and lower PA) would be stronger among those reporting greater AARC-losses, possibly because of AARC-losses reflecting a heightened sensitivity to negative age stereotypes (Wahl et al., 2022). In contrast, we expected associations of COVID-19 disruption with psychosocial outcomes to be weaker among those reporting higher AARC-gains, as perceptions of gains could play a role in supporting effective self-regulatory and coping processes (Diehl & Wahl, 2010; Wilton-Harding & Windsor, 2021).

Finally, we considered the combined effects of AARC-gains and AARC-losses as possible moderators of the associations between COVID-19 disruption and psychosocial outcomes. Negative associations of AARC-losses with psychosocial functioning tend to be weaker among those who perceive relatively higher AARC-gains, suggesting that perceived gains might play a role in off-setting detrimental effects of AARC-losses on psychosocial functioning (Windsor et al., 2022). We therefore tested three-way interactions of COVID-19 disruption × AARC-losses × AARC-gains in the prediction of perceived stress, NA, and PA. We were particularly interested in the possibility that amplifying effects of AARC-losses on the associations of COVID-19 disruption with higher perceived stress and worse affect would become less evident at higher levels of AARC-gains.

## Method

### Study Design and Participants

CloudResearch’s Prime Panels platform (Litman et al., 2017) was used to recruit 318 participants from the United States who completed an online survey via Qualtrics software. We excluded 55 participants; 49 with missing data on one or more key variables, 3 with suspicious response patterns (e.g., endorsing the same response option on a series of unrelated items) or completion times (i.e., under 5 min for an approximately 25-min survey), 2 who were younger than 40, and one who failed 3 (of 3) attention checks. The final sample size was 263, including 56.3% females (43.7% males) aged from 40 to 83 years (*M* = 62.88, standard deviation [*SD*] = 9.00). Our sample consisted of those aged 40 and older as AARC becomes salient around midlife (Brothers, 2016; Wurm & Westerhof, 2015). A majority of the sample was employed (65.4%) and had completed tertiary education (66.2%). In terms of ethnicity, most participants identified as White (86.3%), or Black or African American (6.1%).

Assuming an *R*^2^ of 0.20 based on previous studies (e.g., Knepple Carney et al., 2020) and α = 0.05, power analysis revealed a sample size of 88 or greater would be needed to detect a significant deviation of *R*^2^ from zero with 80% power. Participants were compensated for their time according to their agreement with CloudResearch (approximately USD$1.50). The study was approved by the Flinders University Human Research Ethics Committee (HEL4461-3) and was not preregistered.

### Measures

#### COVID-19 disruption

We developed 25 items to capture aspects of COVID-19 disruption from the onset of the pandemic in March 2020 to July 2021 when the questionnaire was distributed. Item development was informed by qualitative data on the impact of COVID-19 collected as part of a previous study conducted by our research group, and dimensions of the Social Readjustment Rating Scale (Holmes & Rahe, 1967). Participants were asked to consider the extent to which the COVID-19 pandemic and subsequent restrictions caused disruption to their daily life including social restrictions (e.g., “I spent more time alone than I would have liked”), health disruptions to self (e.g., “I have been ill myself as a result of contracting COVID-19”) and others (e.g., “Close relatives or friends of mine have contracted COVID-19”), and disruptions to work (e.g., “I was required to work from home”). Responses were provided on 5-point Likert scales (1 = *not at all true for me* to 5 = *very true for me*).

Principal components analysis revealed that after the exclusion of six cross-loading items, the data were best represented by three dimensions broadly capturing experiences of Social and Lifestyle Disruption (*a* = 0.87; nine items), Work and Health Disruption (*a* = 0.84; eight items), and Others Contracting COVID-19 (*a* = 0.73; two items). Items were summed to produce a separate score for each dimension. Details of the development of the measure are included in Supplementary Materials.

Our measure of COVID-19 disruption shared substantial conceptual overlap with recently published validated measures including the COVID-19 Exposure Index (Hoogendijk et al., 2022) and the COVID-19 Impact Scale (Min et al., 2022); for example, both assess disruptions to out-of-home activity and work arrangements and record impacts on one’s own and other’s health. Unlike the COVID-19 Impact Scale, our measure did not directly tap into psychological effects, reducing potential problems of circularity between our independent and dependent variables (Hahn, 2011). Our use of 5-point rating scales may have also better captured subtle between-person differences in degrees of exposure than is possible with the dichotomous response format of the COVID-19 Exposure Index (Hoogendijk et al., 2022).

#### Awareness of age-related change

AARC was assessed using the AARC-10 Short Form (Kaspar et al., 2019). The measure consists of two five-item subscales assessing AARC-gains and AARC-losses across the domains of health and physical functioning, cognitive functioning, interpersonal relations, social-cognitive and social-emotional functioning, and lifestyle and engagement (Diehl & Wahl, 2010). Participants responded using a 5-point scale ranging from 1 (*not at all*) to 5 (*very much*) to items regarding how their life may have changed as a result of growing older (i.e., “I have a better sense of what is important to me”; “I have less energy”). Scores for each subscale were summed, with high scores reflecting greater AARC-gains (*α* = 0.77) and AARC-losses (*α* = 0.86), respectively.

#### Perceived stress

The Perceived Stress Scale (Cohen et al., 1983) includes 10 items, that ask about one’s experiences of stress over the past month (e.g., “In the last month, how often have you found that you could not cope with all the things you had to do?”). Items were scored on a 5-point Likert scale (0 = *Never* to 4 = *Very Often*). A total summed perceived stress score was computed. Higher scores reflected greater perceived stress. Internal consistency was good, α = 0.93.

#### NA and PA

The Scale of Positive and Negative Experiences was used to measure NA and PA (Diener et al., 2009). Participants were presented with six positive (e.g., “pleasant” and “joyful”) and six negative (e.g., “unpleasant” and “sad”) feeling words and were asked to rate how often they experienced each over the last month. Items were scored on a 5-point Likert scale (0 = *Very rarely or never experienced* to 5 = *Very often or always experienced*). Scores ranged from 6 to 30 on each subset, with higher scores indicative of stronger NA and PA. Good internal consistency was found for both the NA (*α* = 0.92) and PA (*α* = 0.94) subsets.

#### Covariates

Consistent with previous studies examining AARC (e.g., Wilton-Harding & Windsor, 2021) we controlled for age, gender (0 = male, 1= female), education (0 = no tertiary qualification, 1 = tertiary education), employment status (0 = in the labor force, 1 = not in the labor force), and physical functioning. Physical functioning was measured using the Physical Functioning Scale (PF-10), a 10-item subscale of the RAND 36-item Health Survey (Ware et al., 2000). Higher scores indicated greater physical functioning. Subjective socioeconomic status (SES) was measured using an online version of the MacArthur Scale of Subjective Social Status (Adler et al., 2000). Participants were shown a picture of a ladder with 10 rungs and asked to imagine that the ladder represented the SES of people in society. The highest rungs represented high SES while the lowest rungs represented low SES. Participants were then asked to select the rung that best represented their SES. Scores were transformed into a continuous variable ranging from low SES (1) to high SES (10).

#### Analysis

Sequential multiple regressions (with cross-product terms to test interactions) were used to test our hypotheses. An overview is provided in Supplementary Materials.

## Results

Descriptive statistics are reporting in Supplementary Tables 3 and 4. Correlations of note include the strong, negative association between physical functioning and AARC-losses (*r* = −0.73) and the negative (albeit weakly) association between age and AARC-losses. There were no associations between AARC-gains and AARC-losses (*r* = −0.09) and age and physical functioning (*r* = 0.00).

### Associations of COVID-19 Disruption With AARC-Gains and AARC-Losses

Results of the multiple regressions used to examine associations between the three dimensions of COVID-19 disruption and AARC are presented in Table 1. The dimensions of COVID-19 disruption accounted for 7.0% of the variance in AARC-losses, with Work and Health Disruption having the strongest unique association (*sr*^2^ = 0.03). For AARC-gains, the COVID-19 dimensions explained a unique 10% of the variance, with Social and Lifestyle Disruption having the strongest unique association (*sr*^2^ = 0.07). Consistent with predictions, participants reporting greater COVID-19 disruption also reported greater AARC-gains and greater AARC-losses.

**Table 1:** Hierarchical Regression Results: COVID-19 Disruption and AARC-Gains and AARC-Losses (*N* = 263)

	COVID-19 disruption → AARC-losses				COVID-19 disruption → AARC-gains			
	*b*	β	95% CIs	*sr* ^2^	*b*	β	95% CIs	*sr* ^2^
Step 1								
Age	−0.09**	−0.16	[−0.14, −0.04]	0.02	0.02	0.06	[−0.03, 0.08]	0.00
Gender	−0.25	−0.03	[−1.06, 0.56]	0.00	1.33**	0.19	[0.51, 2.19]	0.04
Education	0.66	0.06	[−0.25, 1.57]	0.00	−0.71	−0.10	[−1.65, 0.23]	0.01
Employment	0.26	0.03	[−0.72, 1.24]	0.00	0.16	0.02	[−0.86, 1.17]	0.00
SES	−0.03	−0.01	[−0.28, 0.23]	0.00	0.45**	0.22	[0.18, 0.71]	0.04
Physical functioning	−0.14**	−0.73	[−0.16, −0.12]	0.53	0.01	0.10	[−0.00, 0.03]	0.01
	*R* ^2^ = 0.562, *F* (6, 255) = 54.46, *p* < .001				*R* ^2^ = 0.091, *F* (6, 255) = 4.24, *p* < .001			
Step 2								
Age	−0.05*	−0.10	[−0.11, −0.00]	0.01	0.02	0.05	[−0.03, 0.07]	0.00
Gender	−0.17	−0.02	[−0.93, 0.60]	0.00	1.15**	0.16	[0.31, 1.90]	0.02
Education	0.51	0.05	[−0.33, 1.35]	0.00	−0.73	−0.10	[−1.60, 0.17]	0.01
Employment	1.11*	0.11	[0.14, 2.08]	0.01	0.30	0.03	[−0.74, 1.18]	0.00
SES	−0.17	−0.06	[−0.41, 0.08]	0.00	0.41**	0.21	[0.18, 0.67]	0.03
Physical functioning	−0.12**	−0.64	[−0.14, −0.11]	0.35	0.02*	0.14	[0.02, 0.04]	0.02
Social and Lifestyle Disruption	0.07*	0.12	[0.02, 0.12]	0.01	0.13**	0.32	[0.09, 0.18]	0.07
Work and Health Disruption	0.16**	0.25	[0.09, 0.24]	0.03	0.02	0.04	[−0.06, 0.10]	0.00
Others Contracting COVID-19	0.03	0.02	[−0.10, 0.16]	0.00	−0.02	−0.02	[−0.17, 0.12]	0.00
	*R* ^2^ = 0.632 *R*^2^_change_ = 0.070, *F*_change_(3, 252) = 15.98, *p* < .001				*R* ^2^ = 0.191 *R*^2^_change_ = 0.100, *F*_change_(3, 252) = 10.39, *p* < .001			

*Notes*: β = standardized regression coefficients; AARC = awareness of age-related change; *b* = unstandardized regression coefficients; CI = confidence interval [lower bound, upper bound]; COVID-19 = coronavirus disease 2019; SES = socioeconomic status. Social and Lifestyle Disruption, Work and Health Disruption, and Others Contracting COVID-19 are the three dimensions of the COVID-19 disruption measure. The unique variance explained by each predictor is represented by squared semipartial correlations (*sr*^2^).

*p < .05. **p < .01.

### The Moderating Function of AARC on Associations of COVID-19 Disruption With Psychosocial Outcomes

Results of the moderation analyses are presented in Supplementary Tables 5–7. Here we discuss the results for perceived stress, NA, and PA, in turn. For perceived stress, analysis of main effects showed that higher AARC-losses was associated with higher perceived stress (*b* = 0.97, 95% confidence interval [CI] = [0.74, 1.21], *p* < .01, *sr*^2^ = 0.12), whereas higher AARC-gains was associated with lower perceived stress (*b* = −0.39, 95% CI = [−0.62, −0.17], *p* < .01, *sr*^2^ = 0.02). The COVID-19 disruption dimensions were not associated with perceived stress.

**Figure 1.**
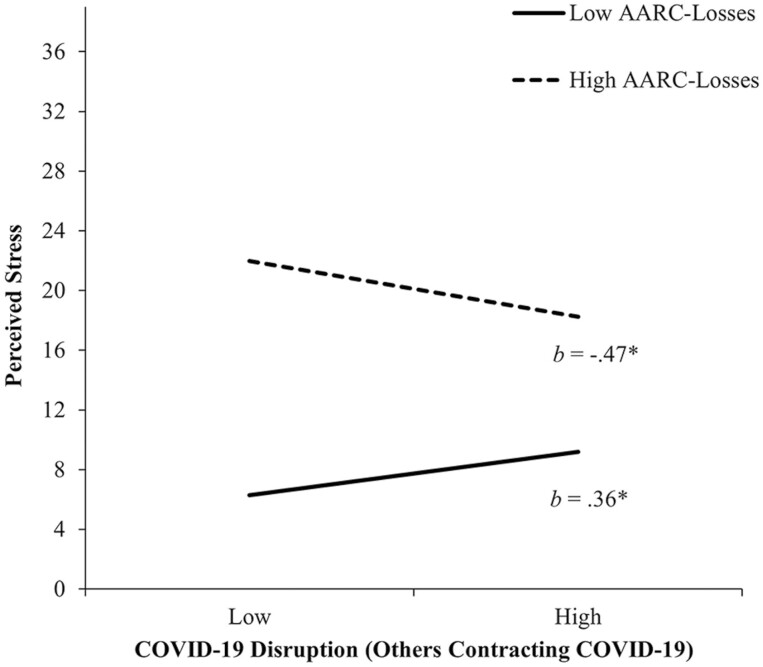
The relationship between COVID-19 disruption (Others Contracting COVID-19) and AARC-losses in predicting perceived stress. High and low values of COVID-19 disruption (Others Contracting COVID-19) and AARC-losses represent 1 *SD* above and below the mean. *b*s represent slopes for COVID-19 disruption (Others Contracting COVID-19) at high and low values of AARC-losses. AARC = awareness of age-related change; COVID-19 = coronavirus disease 2019; *SD* = standard deviation. *p < .05.

A significant interaction between the Others Contracting COVID-19 dimension and AARC-losses accounted for an additional 1.2% of the variance in perceived stress. Figure 1 shows that among participants with lower AARC-losses (−1 *SD*), Others Contracting COVID-19 was positively associated with perceived stress (*b* = 0.36, 95% CI = [0.03, 0.69], *p* = .03). However, among those with higher AARC-losses (+1 *SD*), Others Contracting COVID-19 was negatively associated with perceived stress (*b* = −0.47, 95% CI = [−0.90, −0.04], *p* = .03). Interactions of AARC-gains × AARC-losses (including their interactions with COVID-19 dimensions) were not significant.

**Figure 2.**
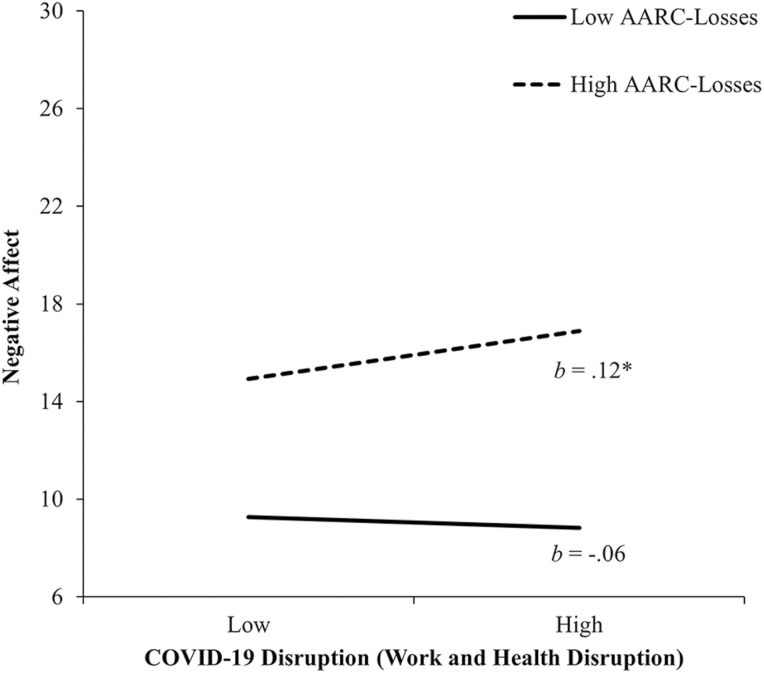
The relationship between COVID-19 disruption (Work and Health Disruption) and AARC-losses in predicting negative affect. High and low values of COVID-19 disruption (Work and Health Disruption) and AARC-losses represent 1 *SD* above and below the mean. *b*s represent slopes for COVID-19 disruption (Work and Health Disruption) at high and low values of AARC-losses. AARC = awareness of age-related change; COVID-19 = coronavirus disease 2019; *SD* = standard deviation. *p < .05.

Analysis of NA showed that higher AARC-losses was associated with greater NA (*b* = 0.55, 95% CI = [0.38, 0.72], *p* < .01, *sr*^2^ = 0.09), whereas higher AARC-gains was associated with lower NA (*b* = −0.26, 95% CI = [−0.42, −0.10], *p* < .01, *sr*^2^ = 0.02). The three dimensions of COVID-19 disruption were not independently associated with NA. A significant interaction between Work and Health Disruption and AARC-losses accounted for an additional 1.9% of the variance in NA. Figure 2 shows that among participants with higher AARC-losses (+1 *SD*), Work and Health Disruption was positively associated with NA (*b* = 0.12, 95% CI = [0.01, 0.22], *p* = .03). However, among those with lower AARC-losses (−1 *SD*), there was no reliable association (*b* = −0.06, 95% CI = [−0.21, 0.08], *p* = .38). Interactions of AARC-gains × AARC-losses and the three-way interactions including COVID-19 dimensions were not significant.

**Figure 3.**
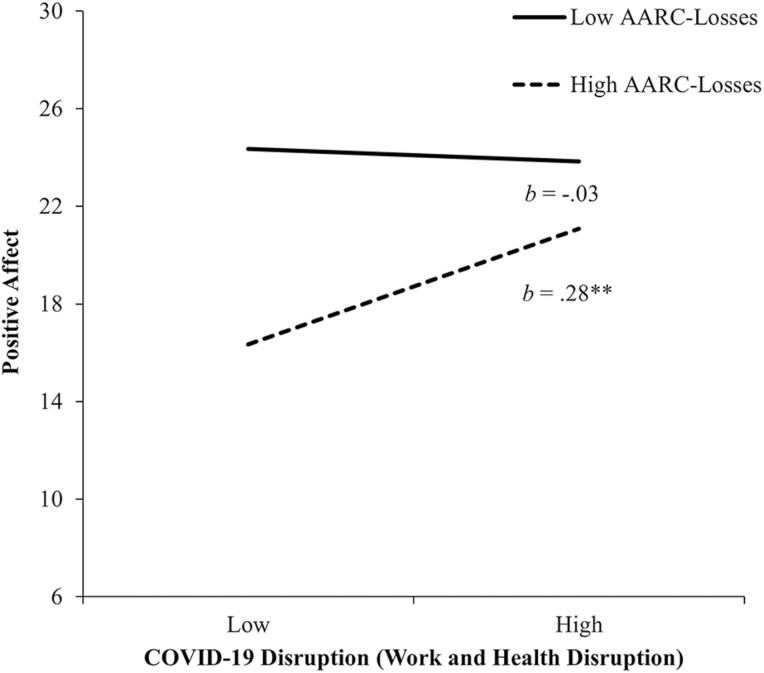
The relationship between COVID-19 disruption (Work and Health Disruption) and AARC-losses in predicting positive affect. High and low values of COVID-19 disruption (Work and Health Disruption) and AARC-losses represent 1 *SD* above and below the mean. *b*s represent slopes for COVID-19 disruption (Work and Health Disruption) at high and low values of AARC-losses. AARC = awareness of age-related change; COVID-19 = coronavirus disease 2019; *SD* = standard deviation. **p < .001.

For PA, higher AARC-losses was associated with lower PA (*b* = −0.46, 95% CI = [−0.62, −0.30], *p* < .01, *sr*^2^ = 0.07), whereas greater AARC-gains was associated with higher PA (*b* = 0.47, 95% CI = [0.31, 0.62], *p* < .01, *sr*^2^ = .09). Work and Health Disruption was positively associated with PA (*b* = 0.16, 95% CI = [0.07, 0.26], *p* < .01, *sr*^2^ = 0.02). A significant interaction between Work and Health Disruption and AARC-losses accounted for an additional 2.9% of the variance in PA. Figure 3 shows that among participants with lower AARC-losses (−1 *SD*), there was no reliable association between Work and Health Disruption and PA (*b* = –0.03, 95% CI = [−0.17, 0.11], *p* = .68). Contrary to expectation, for those with higher AARC-losses (+1 *SD*), Work and Health Disruption was positively correlated with PA (*b* = 0.28, 95% CI = [0.18, 0.38], *p* < .001).

**Figure 4.**
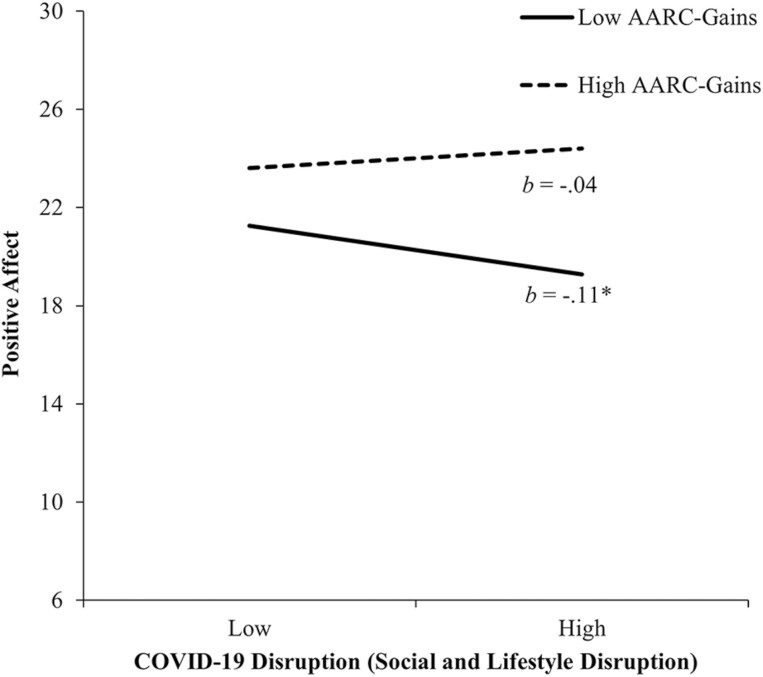
The relationships between COVID-19 disruption (Social and Lifestyle Disruption) and AARC-gains in predicting positive affect. High and low values of COVID-19 disruption (Social and Lifestyle Disruption) and AARC-losses represent 1 *SD* above and below the mean. *b*s represent slopes for COVID-19 disruption (Social and Lifestyle Disruption) at high and low values of AARC-losses. AARC = awareness of age-related change; COVID-19 = coronavirus disease 2019; *SD* = standard deviation. *p < .05.

A significant interaction between Social and Lifestyle Disruption and AARC-gains also accounted for an additional 1.4% of the variance in PA. Figure 4 shows that for those with lower AARC-gains (–1 *SD*), there was a negative association between Social and Lifestyle Disruption and PA (*b* = −0.11, 95% CI = [−0.19, −0.03], *p* = .004). For those experiencing higher AARC-gains (+1 *SD*), there was no reliable association between these two variables (*b* = 0.04, 95% CI = [−0.05, 0.13], *p* = .34). Interactions of AARC-gains × AARC-losses (including their interactions with COVID-19 dimensions) were not significant.

## Discussion

We extended literature on subjective aging by investigating (a) associations of COVID-19 disruption with AARC (gains and losses), and (b) whether AARC moderated associations between dimensions of COVID-19 disruption (Social and Lifestyle Disruption, Work and Health Disruption, Others Contracting COVID-19) and psychosocial outcomes (perceived stress, NA, and PA). Greater Work and Health Disruption was associated with greater AARC-losses. Greater Social and Lifestyle Disruption was associated with greater levels of both AARC-gains and AARC-losses. These associations were statistically reliable after adjustment for age, gender, education, SES, employment status, and physical functioning. We found some evidence of an exacerbating effect of AARC-losses on NA in the context of Work and Health Disruption and a possible protective effect of AARC-gains on PA in the face of Social and Lifestyle Disruption.

### Associations of COVID-19 Disruption and AARC-Gains and AARC-Losses

Our findings are consistent with research reporting greater salience of AARC-losses and AARC-gains in the face of the COVID-19 disruption (Terracciano et al., 2021; Wahl et al., 2022). While our data were cross-sectional and did not provide the opportunity to examine mechanisms, it is possible to speculate about possible underlying processes. On the one hand, negative stereotyping of older adults as vulnerable and most at risk of serious health complications from COVID-19 may increase the salience of AARC-losses for some older adults required to limit out-of-home activity, particularly those with underlying health conditions (Kornadt et al., 2021; Wahl et al., 2022). Others may want to psychologically distance themselves from negative age-related stereotypes and may do so by attending to the characteristics and resources that they perceive as having grown stronger with aging (Terracciano et al., 2021).

An additional plausible explanation for the positive association between Social and Lifestyle Disruption and AARC-gains may be that those experiencing greater AARC-gains are usually more engaged in meaningful activities (Windsor et al., 2022). Therefore pandemic-related restrictions may have resulted in more significant disruption to their lifestyles. Alternatively, those for whom greater AARC-losses reflect objective losses may have found it harder to enact self-­regulatory strategies (Brandtstädter & Rothermund, 2002; Dutt et al., 2018) to adapt to pandemic-related stressors, resulting in greater disruption.

It should also be noted that AARC was unrelated to Others Contracting COVID-19, which may reflect the more contextual, less self-relevant nature of this dimension of disruption (Sabatini et al., 2020). Further, the positive association between social disruption and AARC-gains aligns with research that suggests that transitionary social events (i.e., beginning and end of a romantic relationship) may come with new experiences and self-knowledge that promote AARC-gains (Rupprecht et al., 2022). It may be that adaptation to pandemic-related social life disruptions promoted similar opportunities for self-knowledge, growth, and an increase in AARC-gains. Longitudinal studies should examine how the exposure to significant history-graded, normative age-graded, and nonnormative events influence how one comes to view their own aging.

### The Moderating Function of AARC

Our tests of moderation provided some qualified support for our predictions. While greater AARC-losses was positively associated with perceived stress, a stronger endorsement of disruption related to Others Contracting COVID-19 resulted in greater perceived stress for those with lower AARC-losses and lower perceived stress for those reporting greater AARC-losses. A reason for this unexpected effect may be that for those experiencing greater AARC-losses, seeing others contract but presumably recover from COVID-19 reduced perceived stress. In contrast, those with lower AARC-losses may be psychologically distancing themselves from old age (Terracciano et al., 2021) and therefore when others contract COVID-19 they may become more aware of their age-­related vulnerability, increasing perceived stress. Importantly, however, the Others Contracting COVID-19 dimension was assessed using just two items and effect sizes were small.

AARC-losses resulted in both greater NA and PA in the face of Work and Health Disruption. The exacerbating role of AARC-losses on NA aligns with research suggesting that those experiencing greater AARC-losses may be less likely to mobilize effective coping strategies (Brandtstädter & Rothermund, 2002; Dutt et al., 2018), at least in the context of stressors related to work and health. Greater AARC-losses has also been associated with increased reactivity to daily stressors (Wilton-Harding et al., 2022) and may represent a greater tendency toward negative affectivity (neuroticism) or pessimism that feed into negative views of one’s subjective aging as well as experiences of negative emotions. However, research also supports the possibility that under some circumstances, AARC-losses may serve an adaptive function of better preparing older adults for aging-related losses in the short term (Wolff et al., 2017). This phenomenon may be implicated in the unexpected positive association of COVID-19 Work and Health Disruption with PA among those reporting higher AARC-losses. Alternatively, it is also plausible that some items measuring Work and Health Disruption may not have necessarily been experienced negatively (i.e., “I was required to work from home” might have been enjoyable for some).

Finally, the AARC-losses × Work and Health Disruption interaction in the prediction of PA could represent a vulnerability to low PA among those perceiving greater AARC-losses who also had lower levels of productive engagement with life pre-COVID. Those engaged with life despite higher AARC-losses may be both (a) in a better position to experience PA (perhaps as a result of dispositional optimism or other positive personality traits), and (b) at a relatively greater risk of having their life disrupted due to lockdowns and decreased opportunities for productive engagement. Thus, the unexpected interaction could be an artifact of those with greater AARC-losses, low PA, and low engagement having “less to lose” as a result of the pandemic.

We also found that Social and Lifestyle Disruption was associated with lower PA, but only among those with lower AARC-gains. It may be that those experiencing greater AARC-gains were better able to utilize self-regulation strategies to cope with disruptions to social life (Brandtstädter & Rothermund, 2002; Dutt et al., 2018; Wilton-Harding & Windsor, 2021). Another possibility is that those experiencing high AARC-gains despite high levels of Social and Lifestyle Disruption were those more likely to attribute the disruption not to their aging but to the temporary COVID-19 situation, enabling the maintenance of PA.

Finally, we did not find evidence in support of interactions between AARC-gains and AARC-losses in the prediction of psychosocial outcomes. Although we have reported interactions of gains with losses in some of our recent work (Wilton-Harding et al., 2022; Windsor et al., 2022), the effects have been relatively weak. Although we see continued promise in considering the possible synergies between AARC-gains and AARC-losses, the current findings point to the main effects of the two dimensions being substantially more important in the context of the pandemic than their combined effects.

### Strengths and Limitations

Our contributions included the direct measurement of COVID-19 disruption and the examination of the durability of associations between COVID-19 disruption, AARC, and psychosocial outcomes beyond the first 12 months of the pandemic. However, we did not assess additional personality factors (e.g., optimism and pessimism), and future studies may benefit from establishing associations of variables of interest with AARC independently of such possible confounders. Second, the extent to which AARC may emerge as a moderator across time and among countries (other than the United States) affected differently by the pandemic remains unclear. Third, while we used the AARC short form for brevity, differentiation of subdomains of AARC-losses and AARC-gains was not possible and may be of interest for future research. Fourth, our measure of COVID-19 disruption did not directly assess the death of close network members which may have better distinguished individual differences in degrees of disruption. Finally, the significant interactions accounted for a modest 1%–3% of the variance in the outcomes of interest. Replication with larger, more representative samples (particularly including those in the fourth age who may be more vulnerable to COVID-19 and other stressors; see Charles & Luong, 2013) is needed to establish the replicability and practical significance of findings related to stressor exposure, views on aging, and well-being.

Further, as disruptions associated with COVID-19 encouraged online communication (Dawel et al., 2020), those unable to connect digitally may have been vulnerable and may have experienced greater disruption and associated AARC-losses (Schlomann et al., 2022). Due to the nature of our sampling (online crowdsourcing) we likely missed a significant proportion of vulnerable older adults unfamiliar with, or without access to information communication technology. Indeed, patterns in our data (e.g., low bivariate correlations of age with physical functioning and AARC-losses, see Supplementary Table 3) suggest that our older participants may be higher functioning (and/or our midlife participants lower functioning) than would be expected in the general population.

## Conclusion

Consistent with research suggesting a greater salience of subjective aging brought about by the pandemic (Terracciano et al., 2021; Wahl et al., 2022), our findings showed that aspects of COVID-19 disruption, particularly Social and Lifestyle Disruption, were reliably associated with both higher AARC-losses and AARC-gains. Results of moderation analysis were less consistent but suggested a possible exacerbating effect of AARC-losses in the face of Work and Health Disruption undermining NA and a possible protective effect of AARC-gains in the context of Social and Lifestyle Disruption undermining PA. Longitudinal research tracking individuals across major life transitions and history-graded influences is needed to better inform whether—and how—subjective aging might be targeted in developing interventions to support late life adaptation.

## Supplementary Material

gbad093_suppl_Supplementary_Materials

## Data Availability

Data and study materials will be made available upon reasonable request to the first author. This study was not preregistered.
