# Supplementary material for: Does Awareness of Aging Matter? The Moderating Function of Awareness of Age-Related Change on the Relationships Between COVID-19 Disruption, Perceived Stress, and Affect
Source: J Gerontol B Psychol Sci Soc Sci. 2023 Jun 20;78(10):1691–9. doi: 10.1093/geronb/gbad093 (PMC10561889; doi:10.1093/geronb/gbad093)
Supplement: gbad093_suppl_Supplementary_Materials [file gbad093_suppl_supplementary_materials.docx]

**Does Awareness of Aging Matter?**

**The Moderating Function of Awareness of Age-Related Change (AARC) on the Relationships Between COVID-19 Disruption, Perceived Stress and Affect.**

Elli Kolovos, BPsych(Hons)

Flinders University, Adelaide, Australia

Tim D. Windsor, Ph.D

Flinders University, Adelaide, Australia

Address correspondence to: Elli Kolovos, College of Education, Psychology and Social Work, Flinders University, GPO Box 2100, Adelaide SA 5001, Australia. Email: [elli.kolovos@flinders.edu.au](mailto:elli.kolovos@flinders.edu.au)

**Supplementary Materials**

**Exploratory Factor Analysis of the COVID-19 Disruption Measure**

We conducted principal-components analyses (varimax rotation) on the 25-item COVID-19 disruption measure. The initial analysis revealed a five-factor solution (based on eigenvalues > 1.0). However, a closer examination of the Scree plot (Cattell, 1978) and factor loadings revealed several poor performing items and significant cross-loadings of items across factors. Howard (2015) recommends that satisfactory items should (1) load onto their primary factor above 0.40, (2) load onto alternate factors below 0.30, and (3) demonstrate a difference of 0.20 between their primary and alternative factor loadings. A subsequent analysis assuming a three-factor solution resulted in fewer cross-loading items and accounted for 53.9% of the variance. We then identified items that had loadings of greater than 0.30 on two or more factors and progressively removed items with the smallest difference in loadings (see Table 2 for the removed items).

Our final COVID-19 disruption measure comprised 19 items that assess three dimensions of disruption. Factor 1 (Social and Lifestyle Disruption) includes nine items that capture the role of COVID-19 restrictions in heightening social isolation, risk assessments and safety monitoring and disrupting usual routines. Factor 2 (Work and Health Disruption) includes eight items that capture disruptions to work and income as well as disruptions resulting from contracting COVID-19. Factor 3 (Others Contracting COVID-19) includes two items that ask respondents if their relatives, friends and acquaintances have contracted COVID-19. While the inclusion of a factor with only two representative items is a limitation, this factor was retained as the items had strong loadings (greater than 0.80) and made conceptual sense. See supplementary Table 1 for item wording and standardised factor loadings for the 19-item scale.

**Supplementary Table 1**

Factor loadings from an exploratory three-dimensional item factor analysis of the 19-item COVID-19 disruption measure.

| Factor | Item No. | Item | Loading |
| --- | --- | --- | --- |
| *As a result of restrictions resulting from COVID-19, and/or my own concerns about transmission of the virus:* | | | |
| F1 (Social and Lifestyle Disruption) | 6 | I spent more time indoors in my house than I would have liked. | .763 |
|  | 8 | I spent less time with loved ones, relatives, friends or acquaintances than I would have liked. | .739 |
|  | 9 | I was unable to leave my house at times when I wanted to. | .733 |
|  | 24 | Disruptions to my life resulting from COVID-19 occurred over a long period of time since March 2020. | .665 |
|  | 7 | I spent more time alone than I would have liked. | .660 |
|  | 23 | My life has been generally disrupted as a result of COVID-19. | .658 |
|  | 16 | I spent a lot of my time keeping up with information about COVID-19 in the media. | .645 |
|  | 11 | I regularly assessed risk and safety when I wanted to leave the house. | .638 |
|  | 14 | I was unable to engage in some of my usual routines, hobbies or interests. | .632 |
| F2  (Work and Health Disruption) | 21 | Working from home was more difficult than working in my usual workplace. | .827 |
|  | 19 | I had to take time off of work or postpone other commitments due to being tested for COVID-19. | .740 |
|  | 18 | I needed to change my usual routine in order to support members of my family (e.g., help with home schooling or a family business). | .726 |
|  | 22 | I had to adapt to a different way of doing things at work. | .690 |
|  | 17 | I experienced a loss of income during COVID-19. | .673 |
|  | 20 | I was required to work from home. | .627 |
| *Since the beginning of the COVID-19 pandemic:* | | | |
|  | 1 | I have been ill myself as a result of contracting COVID-19. | .667 |
|  | 4 | I have reduced my normal amount of exercise because of restrictions or concerns about COVID-19. | .477 |
| F3 (Others contracting COVID-19) | 3 | People who I knew but am not close to have contracted COVID-19 | .846 |
|  | 2 | Close relatives or friends of mine have contracted COVID-19 | .812 |
|  |  |  |  |
|  |  |  |  |

*Note.* Items 5, 10, 12, 13, 15, 25 were removed from the final 19-item COVID-19 disruption measure. General instructions for the measure were as follows, “The coronavirus (COVID-19) was declared a global pandemic by the World Health Organisation on the 11th of March 2020. Since then, health problems related to the virus as well as restrictions placed on movement and travel have resulted in disruptions to many peoples’ lives. We are interested in the extent to which the COVID-19 pandemic has created disruptions to your life between March 2020 and now. Please respond to the following statements on a scale of 1 (not at all true for me) to 5 (very true for me)”.

**Supplementary Table 2**

Items that were excluded from the 19-item COVID-19 measure for loading above 0.30 on two or more factors.

| Item No. | Excluded Items |
| --- | --- |
|  | *Since the beginning of the COVID-19 pandemic:* |
| 5 | My diet has been less healthy than what it normally is because of restrictions or concerns about COVID-19. |
|  | *As a result of restrictions resulting from COVID-19, and/or my own concerns about transmission of the virus:* |
| 10 | I was asked to quarantine for a period of time mandated by my government. |
| 12 | I had more difficulty accessing food and other essential items than normal |
| 13 | I had to postpone or rearrange travel plans |
| 15 | I was unable to attend an event that was important to me (i.e., wedding, birthday, graduation, funeral) |
| 25 | I continue to experience disruptions to my life resulting from COVID-19 |

**Statistical Analyses and Considerations**

The data met statistical assumptions. Histograms were used to assess normality and the absence of outliers across outcome variables. Scatterplots also confirmed no serious deviations from normality. Homoscedasticity was assessed by scatterplots of standardised residuals against standardised predicted values, with data well distributed across all outcome variables. Finally, the absence of multicollinearity was confirmed by examining variance inflation factor (VIF) values. Values were well below 5 across all outcome variables suggesting that separate constructs had been captured. Ultimately, assumptions for normality, homoscedasticity and multicollinearity were met.

To examine means and bivariate relationships, we computed descriptive statistics and correlations for the variables of interest. To assess whether greater COVID-19 disruption was associated with greater AARC-losses and AARC-gains, hierarchical regressions were run across each outcome variable using IBM SPSS 26. All covariates (age, gender, education, employment, SES, physical functioning) were entered at Step 1 and each dimension of COVID-19 disruption (Social and Lifestyle Disruption, Work and Health Disruption, Others Contracting COVID-19) was entered at Step 2.

Multiple hierarchical regressions were also used to examine the moderating function of AARC (gains and losses). Each dimension of COVID-19 disruption, AARC-gains and AARC-losses were mean centred, and cross-product terms were computed to test interactions. Models were specified over several sequential steps across each outcome variable. All covariates were entered at Step 1 and the relevant predictors and/or interaction terms were entered in subsequent steps. The unique variance explained by each predictor was represented by squared semi-partial correlations (sr^2^ x 100 = % variance explained). We first tested the highest order interactions and progressively entered and excluded interaction terms that were nonsignificant allowing us to arrive at a final model.  Significant interactions were further probed via tests of simple slopes and plotted using the PROCESS 4.0 SPSS macro (Hayes, 2017).

**Supplementary Table 3**

Descriptive Statistics for Key Variables (*N* = 263)

| Range | | | | |
| --- | --- | --- | --- | --- |
| Variable (*n*) | *M/%* | *SD* | Min | Max |
| Gender (female %) | 56.30 |  |  |  |
| Ethnicity (white %) | 86.30 |  |  |  |
| Education (tertiary %) | 66.20 |  |  |  |
| Employment (employed %) | 65.40 |  |  |  |
| Age | 62.88 | 9.00 | 40 | 83 |
| Socioeconomic Status | 5.34 | 1.75 | 1 | 10 |
| Physical Functioning | 73.67 | 25.52 | 0 | 100 |
| Social and Lifestyle Disruption | 30.71 | 8.49 | 9 | 45 |
| Work and Health Disruption | 16.37 | 7.71 | 8 | 38 |
| Others Contracting COVID-19 | 6.16 | 2.96 | 2 | 10 |
| Awareness of Age-Related Losses (AARC Losses) | 12.22 | 4.89 | 5 | 25 |
| Awareness of Age-Related Gains  (AARC Gains) | 19.97 | 3.51 | 7 | 25 |
| Perceived Stress | 13.42 | 8.34 | 0 | 39 |
| Negative Affect | 12.79 | 5.41 | 6 | 30 |
| Positive Affect | 22.04 | 4.99 | 7 | 30 |

*Note.* The three dimensions of the COVID-19 disruption measure include Social and Lifestyle Disruption, Work and Health Disruption and Others Contracting COVID-19.

**Supplementary Table 4**

Correlation coefficients among key predictors and covariates (*n* = 263)

|  | 1. | 2. | 3. | 4. | 5. | 6. | 7. | 8. | 9. | 10. | 11. | 12. | 13. | 14. | 15. |
| --- | --- | --- | --- | --- | --- | --- | --- | --- | --- | --- | --- | --- | --- | --- | --- |
| 1. Gender ^a^ | - | .02 | -.08 | .11 | .10 | -.08 | .01 | .09 | -.16** | .00 | -.05 | .19** | .02 | .04 | -.01 |
| 2. Ethnicity ^b^ |  | - | .03 | .04 | -.02 | -.05 | -.05 | .05 | .02 | .04 | .02 | .07 | -.01 | .06 | -.04 |
| 3. Education ^c^ |  |  | - | -.12* | -.20** | .36** | -.01 | .01 | .22** | .05 | .10 | -.05 | .12 | .01 | .12* |
| 4. Employment ^d^ |  |  |  | - | .51** | -.26** | -.00 | -.01 | -.51** | -.12* | -.07 | .03 | -.05 | -.07 | -.08 |
| 5. Age |  |  |  |  | - | -.27** | .00 | .01 | -.49** | -.16* | -.16* | .05 | -.21** | -.00 | -.22** |
| 6. SES |  |  |  |  |  | - | .06 | .02 | .34** | .05 | .01 | .16* | -.13* | .39** | -.11 |
| 7. Physical Functioning |  |  |  |  |  |  | - | -.08 | -.28** | -.05 | -.73** | .18 | -.51** | .24** | -.42** |
| 8. Social and Lifestyle Disruption^e^ |  |  |  |  |  |  |  | - | .34** | .32** | .25** | .33** | .21** | .03 | .19** |
| 9. Work and Health Disruption^f^ |  |  |  |  |  |  |  |  | - | .28** | .47** | .07 | .35** | .10 | .33** |
| 10. Others Contracting COVID-19^g^ |  |  |  |  |  |  |  |  |  | - | .16** | .07 | .13* | .07 | .14* |
| 11. AARC Losses |  |  |  |  |  |  |  |  |  |  | - | -.09 | .69** | -.34** | .60** |
| 12. AARC Gains |  |  |  |  |  |  |  |  |  |  |  | - | -.21** | .42** | -.20** |
| 13. Perceived Stress |  |  |  |  |  |  |  |  |  |  |  |  | - | -.60** | .79** |
| 14. Positive Affect |  |  |  |  |  |  |  |  |  |  |  |  |  | - | -.58** |
| 15. Negative Affect |  |  |  |  |  |  |  |  |  |  |  |  |  |  | - |

*Notes.* *p < .05, ** p < .01, a 0= male, 1= female, b 0= white, 1= other, c 0= no tertiary qualification, 1= tertiary qualification, d 0= in labour force, 1= not on labour force, _e, f, g_ = COVID-19 disruption dimensions.

|  | Step 2 | | | | | Step 3 | | | | |
| --- | --- | --- | --- | --- | --- | --- | --- | --- | --- | --- |
|  | *b* | *β* | *95% CIs* | Sr^2^ | | | *b* | *β* | 95% CIs | Sr^2^ |
| Age | -.13* | -.14 | [-.23, -.03] | .01 | | | -.11* | -.11 | [-.21, -.01] | .01 |
| Gender | 1.37 | .08 | [-.10, 2.85] | .01 | | | 1.14 | .07 | [-.33, 2.62] | .00 |
| Education | 1.53 | .09 | [-.09, 3.15] | .01 | | | 1.75* | .10 | [.14, 3.36] | .01 |
| Employment | .99 | .06 | [-.89, 2.86] | .00 | | | .83 | .05 | [-1.03, 2.68] | .00 |
| SES | -.86** | -.18 | [-1.33, -.39] | .02 | | | -.85** | -.18 | [-1.31, -.39] | .02 |
| Physical Functioning | -.01 | -.03 | [ -.05, .03] | .00 | | | .00 | .00 | [-.04, .04] | .00 |
| Social and Lifestyle Disruption | .09 | .09 | [-.02, .19] | .01 | | | .07 | .07 | [-.03, .18] | .00 |
| Work and Health Disruption | .08 | .08 | [-.06, .23] | .00 | | | .12 | .12 | [-.03, .26] | .00 |
| Others Contracting COVID-19 | -.02 | -.01 | [-.28, .24] | .00 | | | -.09 | -.03 | [-.35, .17] | .00 |
| AARC Losses | .97** | .57 | [.74, 1.21] | .12 | | | .99** | .58 | [.76, 1.23] | .12 |
| AARC Gains | -.39** | -.17 | [-.62, -.17] | .02 | | | -.35** | -.15 | [-.57, -.13] | .02 |
| Others Contracting COVID-19*Losses |  |  |  |  | | | -.07* | -.12 | [-.12, -.02] | .01 |
|  | *R^2^ = .543*  *R^2^_change_* = .203, *F_change_* (5, 250) = 22.26, *p* <.001. | | | | *R^2^ = .555*  *R^2^*_change_ = .012, *F_change_* (1, 249) = 6.78, *p* = .01. | | | | | |

**Supplementary Table 5**

Hierarchical Regression Results: Perceived Stress (*N* = 263)

*Note.* Age, gender, education, employment status, SES, and Physical Functioning were entered at Step 1, accounting for an initial 34.0% of the variance. CI= confidence interval [lower bound, upper bound]; AARC= awareness of age-related change. *b* = unstandardised regression coefficients, *β* = standardised regression coefficients. Social and Lifestyle Disruption, Work and Health Disruption, and Others Contracting COVID-19 are the three dimensions of the COVID-19 disruption measure. The unique variance explained by each predictor is represented by squared semi-partial correlations (Sr^2^).

*p < .05. **p <.01.

**Supplementary Table 6**

Hierarchical Regression Results: Negative Affect (*N* = 263)

|  | Step 2 | | | | | Step 3 | | | | |
| --- | --- | --- | --- | --- | --- | --- | --- | --- | --- | --- |
|  | *b* | *β* | *95% CIs* | Sr^2^ | | | *b* | *β* | 95% CIs | Sr^2^ |
| Age | -.08* | -.13 | [-.15, -.00] | .01 | | | -.07* | -.12 | [-.14, -.00] | .01 |
| Gender | .64 | .06 | [-.43, 1.70] | .00 | | | .65 | .06 | [-.40, 1.70] | .00 |
| Education | 1.05 | .09 | [-.12, 2.21] | .01 | | | 1.01 | .09 | [-.14, 2.15] | .01 |
| Employment | .38 | .03 | [-.97, 1.73] | .00 | | | .05 | .01 | [-1.30, 1.40] | .00 |
| SES | -.56** | -.18 | [-.89, -.22] | .02 | | | -.56** | -.18 | [-.90, -.23] | .02 |
| Physical Functioning | .00 | .01 | [-.03, .03] | .00 | | | .00 | .02 | [-.03, .03] | .00 |
| Social and Lifestyle Disruption | .06 | .09 | [-.02, .13] | .00 | | | .07 | .11 | [-.01, .14] | .01 |
| Work and Health Disruption | .06 | .09 | [-.04, .17] | .00 | | | .00 | .00 | [-.11, .11] | .00 |
| Others Contracting COVID-19 | .01 | .01 | [-.18, .19] | .00 | | | .01 | .00 | [-.17, .18] | .00 |
| AARC Losses | .55** | .50 | [.38, .72] | .09 | | | .55** | .50 | [.38, .72] | .09 |
| AARC Gains | -.26** | -.17 | [-.42, -.10] | .02 | | | -.29** | -19 | [-.45, -.13] | .03 |
| Work and Health Disruption*Losses |  |  |  |  | | | .02** | .16 | [.01, .03] | .02 |
|  | *R^2^ =* .429  *R^2^_change_* = .173 *F_change_* (5, 250) = 15.14, *p* <.001. | | | | *R^2^ =* .448  *R^2^*_change_ = .019, *F_change_* (1, 249) = 8.67, *p* = .004. | | | | | |

*Note.* Age, gender, education, employment status, SES, and Physical Functioning were entered at Step 1, accounting for an initial 25.6% of the variance. CI= confidence interval [lower bound, upper bound]; AARC= awareness of age-related change. *b* = unstandardised regression coefficients, *β* = standardised regression coefficients. Social and Lifestyle Disruption, Work and Health Disruption, and Others Contracting COVID-19 are the three dimensions of the COVID-19 disruption measure. The unique variance explained by each predictor is represented by the squared semi-partial correlations (Sr^2^).

*p < .05. **p <.01.

**Supplementary Table 7**

Hierarchical Regression Results: Positive Affect (*N* = 263)

|  | Step 2 | | | | | Step 3 | | | | |
| --- | --- | --- | --- | --- | --- | --- | --- | --- | --- | --- |
|  | *b* | *β* | *95% CIs* | Sr^2^ | | | *b* | *β* | 95% CIs | Sr^2^ |
| Age | .05 | .09 | [-.02, .12] | .01 | | | .05 | .08 | [-.02, .11] | .00 |
| Gender | .17 | .51 | [-.83, 1.16] | .00 | | | .06 | .01 | [-.90, 1.02] | .00 |
| Education | -.98 | -.09 | [-2.07, .11] | .01 | | | -1.02 | -.10 | [-2.07, .02] | .01 |
| Employment | .51 | .05 | [-.76, 1.77] | .00 | | | -.11 | -.01 | [-1.35, 1.13] | .00 |
| SES | .95** | .33 | [.64, 1.27] | .08 | | | .93** | .32 | [.63, 1.23] | .08 |
| Physical Functioning | -.02 | -.08 | [-.04, .01] | .00 | | | -.01 | -.07 | [-.04, .01] | .00 |
| Social and Lifestyle Disruption | -.05 | -.09 | [-.12, .02] | .01 | | | -.03 | -.05 | [.10, .04] | .00 |
| Work and Health Disruption | .16** | .25 | [.07, .26] | .02 | | | .08 | .13 | [-.02, .18] | .01 |
| Others Contracting COVID-19 | .13 | .08 | [-.04, .30] | .00 | | | .13 | .08 | [-.04, .29] | .00 |
| AARC Losses | -.46** | .45 | [-.62, -.30] | .07 | | | -.47** | -.46 | [-.62, -.31] | .08 |
| AARC Gains | .47** | .33 | [.31, .62] | .09 | | | .46** | .33 | [.32, .61] | .08 |
| Work and Health Disruption*Losses |  |  |  |  | | | .02** | .20 | [.01, .04] | .03 |
| Social and Lifestyle Disruption*Gains |  |  |  |  | | | .02* | .12 | [.00, .03] | .01 |
|  | *R^2^ =* .425  *R^2^_change_* = .192, *F_change_* (5, 250) = 16.72, *p* <.001. | | | | *R^2^ =* .472  *R^2^*_change_ = .046, *F_change_* (2, 248) = 10.89, *p* <.001. | | | | | |

*Note.* Age, gender, education, employment status, SES, and Physical Functioning were entered at Step 1, accounting for an initial 23.3% of the variance. CI= confidence interval [lower bound, upper bound]; AARC= awareness of age-related change. *b* = unstandardised regression coefficients, *β* = standardised regression coefficients. Social and Lifestyle Disruption, Work and Health Disruption, and Others Contracting COVID-19 are the three dimensions of the COVID-19 disruption measure. The unique variance explained by each predictor is represented by the squared semi-partial correlations (Sr^2^).

*p < .05. **p <.01.

**References**

Cattell, R.B. (1978). Conducting a Factor Analytic Research: Strategy and Tactics. In: The Scientific Use of Factor Analysis in Behavioral and Life Sciences. Springer, Boston, MA. https://doi.org/10.1007/978-1-4684-2262-7_15

Hayes, A. F. (2017). Introduction to mediation, moderation, and conditional process analysis: a regression-based approach. Guilford Publications.

Howard, M. C. (2016). A review of exploratory factor analysis decisions and overview of current practices: What we are doing and how can we improve?. *International Journal of Human-Computer Interaction*, *32*(1), 51-62. https://doi.org/10.1080/10447318.2015.1087664
